# Activation of Cannabinoid Receptor 1 Enhances Wound Healing by Promoting the Proliferative Phase

**DOI:** 10.3390/ijms27031171

**Published:** 2026-01-23

**Authors:** Hui Song Cui, Ya Xin Zheng, Yoon Soo Cho, Yeon Gyun Jung, In Suk Kwak, Yu Mi Ro, So Young Joo, June-Bum Kim, Cheong Hoon Seo

**Affiliations:** 1 Burn Institute, Department of Rehabilitation Medicine, Hangang Sacred Heart Hospital, College of Medicine, Hallym University, 94-200 Yeongdeungpo-Dong, Yeongdeungpo-Ku, Seoul 07247, Republic of Korea; bioeast@hanmail.net (H.S.C.); yxzheng2023@gmail.com (Y.X.Z.); j1076@naver.com (Y.G.J.); nym8060@hanmail.net (Y.M.R.); 2 Department of Rehabilitation Medicine, Hangang Sacred Heart Hospital, College of Medicine, Hallym University, 94-200 Yeongdeungpo-Dong, Yeongdeungpo-Ku, Seoul 07247, Republic of Korea; hamays@hanmail.net (Y.S.C.); anyany98@naver.com (S.Y.J.); 3 Department of Anesthesiology and Pain Medicine, Hangang Sacred Heart Hospital, College of Medicine, Hallym University, 94-200 Yeongdeungpo-Dong, Yeongdeungpo-Ku, Seoul 07247, Republic of Korea; 031132@hallym.or.kr; 4 Medical Genetics Clinic, Uijeongbu Eulji Medical Center, College of Medicine, Eulji University, Uijeongbu 11759, Republic of Korea

**Keywords:** cannabinoid receptor 1, wound healing, fibroblasts, SMAD, non-SAMD

## Abstract

The mechanisms underlying wound healing mediated by cannabinoid receptor 1 (CB1)—known for its neuromodulatory functions—remain incompletely understood. Therefore, we investigated the impact of activating CB1 using specific agonists, both in vitro and in vivo, with a focus on wound healing. In the in vitro study, fibroblasts were isolated and cultured from the dermis of human skin and treated with a CB1 agonist, 2-arachidonyl glyceryl ether (2-AGE). In the in vivo study, a mouse acute wound model was created using a skin biopsy punch and treated with the CB1 agonist arachidonoyl 2′-chloroethylamide (ACEA). The in vitro study revealed that 2-AGE increased cell proliferation and differentiation, upregulated the expression of alpha-smooth muscle actin (α-SMA), N-cadherin, and vimentin, and enhanced cell migration as well as the synthesis of type I and III collagen and fibronectin in normal human dermal fibroblasts. The CB1 antagonist AM251 abolished 2-AGE-induced expression of α-SMA, type I collagen, and fibronectin. In vivo, ACEA treatment accelerated wound closure, increased expression of α-SMA, type I collagen, and fibronectin, and ultimately increased epidermal and dermal thickness. Overall, these findings suggest that the activation of CB1 promotes wound healing and provides evidence for the therapeutic potential of CB1 agonists in wound treatment.

## 1. Introduction

The natural healing of cutaneous wounds is a dynamic and multifaceted biological process involving distinct and overlapping stages of inflammation, proliferation, and remodeling [1]. Among the key cellular contributors to wound healing, dermal fibroblasts play a critical role in high-quality tissue repair. Notably, at the end of the inflammatory stage, these fibroblasts migrate from the wound edge to the wound site via chemo-attractants and in response to multiple growth factors, including transforming growth factor beta 1 (TGF-β1), activin, and bone morphogenetic protein (BMP). These signals stimulate fibroblast proliferation and differentiation into myofibroblasts, which mediate wound contraction, reduce wound size, and produce collagen and other extracellular matrix (ECM) components, through the fibroblast-to-myofibroblast transition (FMT). This activity contributes to the formation of granulation tissue and scar formation, thereby supporting wound repair [1].

FMT involves multiple signal transduction pathways, with the most widely recognized being Small Mothers Against Decapentaplegic (SMAD)-dependent and SMAD-independent (non-SMAD) signaling pathways [2]. The SMAD pathway is a primary signaling cascade readily activated by TGF-β1, which plays a vital role in normal wound healing and contributes to the pathological mechanisms underlying dermal proliferative diseases, such as hypertrophic scars (HTS). Phosphorylated SMAD2 and SMAD3 are associated with SMAD4, forming a transcriptionally active complex and then into the nucleus, regulating gene expression. Non-SMAD signaling pathway, including TGF-β1, activated kinase 1 (TAK1), which downstream Jun terminal kinase (JNK), extracellular signal-regulated kinase 1/2 (ERK1/2), and p38 MAPK, as well as protein kinase B (AKT) and nuclear factor kappa-light-chain-enhancer of activated B cells (NF-κB) signaling [3,4]. Dysregulation of fibroblast activity can result in chronic wounds or pathological scarring, underscoring the need for targeted therapeutic strategies [5].

Recent research has highlighted the role of the endocannabinoid system (ECS) in skin physiology and repair. The cannabinoid receptor 1 (CB1), a G protein-coupled receptor abundantly expressed in the nervous system, is primarily known for its neuromodulatory functions, and it is also expressed in peripheral tissues, including skin tissues [6]. An in vitro study reported that porcine primary fibroblasts treated with delta-9 tetrahydrocannabinol (THC), a natural phytocannabinoid, improved cell migration and accelerated wound closure [7]. An in vivo study showed that CB1 activation was observed in inflammation, proliferation, and remodeling phases of the wound healing process [8]. Moreover, CB1 deficiency prevented bleomycin-induced dermal fibrosis, whereas CB1 agonist arachidonoyl-chloro-ethanolamide (ACEA) increased leukocyte infiltration, enhanced dermal thickness, and elevated the number of myofibroblasts when mice were treated with bleomycin [9]. Clinical evidence indicates that the topical application of Cannabis-Based Medicines (TCBMs) facilitates tissue repair and promotes complete wound closure in previously refractory wounds [10]. This compelling clinical efficacy suggests a strong potential for cannabinoid-based wound management strategies.

Despite these promising findings, the mechanisms underlying CB1-mediated wound healing are not yet fully understood. Consequently, we hypothesize that the observed beneficial clinical outcome is partially mediated by the activation of the CB1 pathway. Our objective was to elucidate the role of CB1 activation in dermal fibroblast function and wound repair, integrating both in vitro cellular assays and in vivo animal experiments. By dissecting the molecular and cellular responses to CB1 stimulation, we sought to establish a foundation for cannabinoid-based therapies in wound healing.

## 2. Results

### 2.1. CB1 Activation Induces Proliferation and Differentiation in Human Dermal Fibroblasts (HDFs)

**Figure 1:**
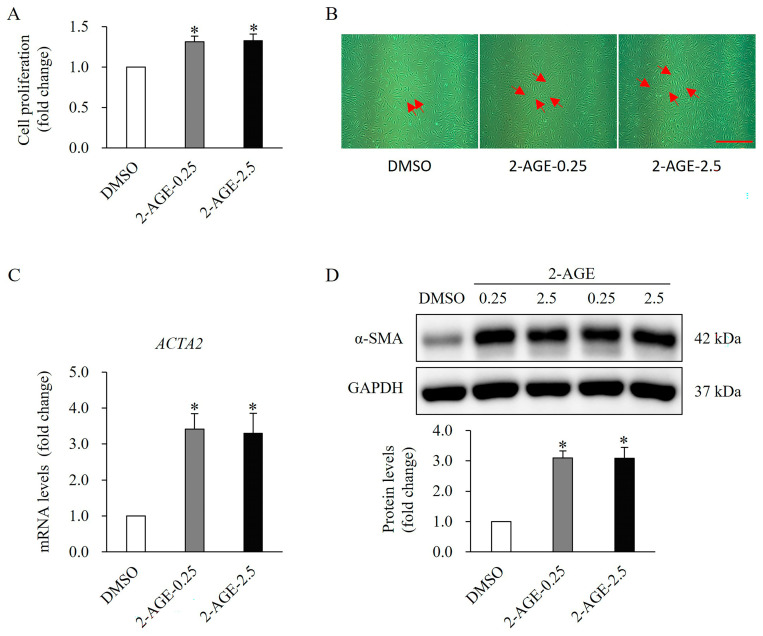
Effect of cannabinoid receptor 1 (CB1) agonist on the proliferation and differentiation of human dermal fibroblasts (HDFs). (**A**) Significant increase in the proliferation of HDFs treated with 0.25 or 2.5 μM 2-AGE, a CB1-selective agonist, for 48 h compared to dimethyl sulfoxide (DMSO)-treated cells. (**B**) Images (10× magnification) showing increased cellularity in HDFs. The red arrow represents proliferating cells. Scale bar, 50 μm. (**C**) Real-time polymerase chain reaction analysis showing a significant increase in mRNA levels in HDF treated with 0.25 or 2.5 μM 2-arachidonyl glyceryl ether (2-AGE), a CB1-selective agonist, for 48 h compared with DMSO-treated control cells. (**D**) Western blot analysis showing a significant increase in alpha-smooth muscle actin protein levels, a differentiation marker, in HDFs treated with 2-AGE for 48 h compared with DMSO-treated cells. Data are presented as mean ± SD; *n* = 3 (each group). * *p* < 0.05 vs. DMSO-treated control cells, which were set as 1.0.

To determine whether CB1 activation affects cellular activity, we conducted cell proliferation assays using the CellTiter-Glo Luminescent Cell Viability Assay Kit. HDFs were treated with the CB1-selective agonist 2-arachidonyl glyceryl ether (2-AGE) at 0.25 or 2.5 μM for 48 h. The proliferation of HDFs significantly increased following treatment with 2-AGE at low concentrations of 0.25 μM and high concentrations of 2.5 μM compared to dimethyl sulfoxide (DMSO)-treated control cells (*p* < 0.05, Figure 1A,B). Notably, no significant differences were observed between the low and high concentrations.

To further investigate the impact of the agonist on cellular differentiation, α-smooth muscle actin (α-SMA), a well-established differentiation marker for dermal fibroblasts, was analyzed using real-time polymerase chain reaction (PCR) and Western blotting. The mRNA and protein levels of αSMA were markedly upregulated following treatment with 2-AGE for 48 h, compared with DMSO-treated cells (*p* < 0.05, Figure 1C,D). Similarly, no significant differences were detected between low and high concentrations.

**Figure 2:**
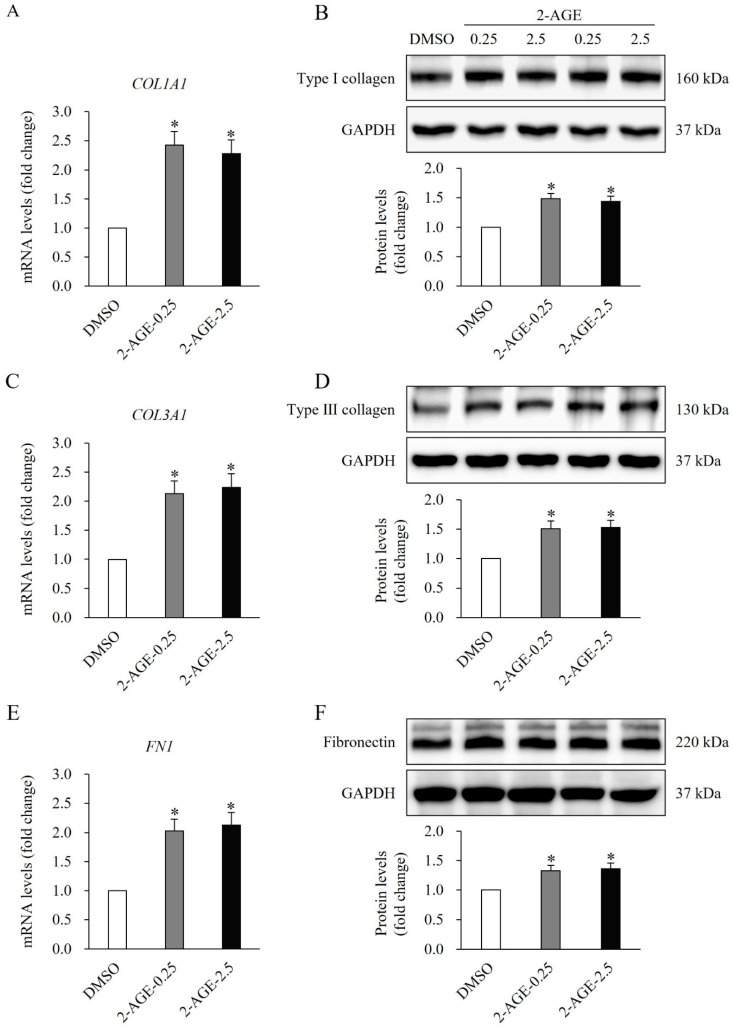
Effects of cannabinoid receptor 1 (CB1) agonist on the extracellular matrix (ECM) synthesis of HDFs. Real-time polymerase chain reaction analysis showing increased mRNA levels of (**A**) type I collagen (*COL1A1*), (**C**) type III collagen (*COL3A1*), and (**E**) fibronectin (*FN1*) in human dermal fibroblasts (HDFs) treated with 0.25 or 2.5 μM 2-arachidonyl glyceryl ether (2-AGE), a CB1-selective agonist, for 48 h compared with dimethyl sulfoxide (DMSO)-treated control cells. Western blot analysis showing increased protein levels of (**B**) type I collagen, (**D**) type III collagen, and (**F**) fibronectin in HDFs treated with 0.25 or 2.5 μM 2-AGE for 48 h compared with DMSO-treated cells. Data are presented as mean ± SD, *n* = 3 per group. * *p* < 0.05 vs. DMSO-treated controls, which were set to 1.0.

The impact of the agonist on ECM synthesis in HDFs was examined by analyzing the expression of key ECM components, including type I collagen, type III collagen, and fibronectin. Treatment with 2-AGE at 0.25 or 2.5 μM for 48 h resulted in a significant increase in the mRNA and protein levels of type I collagen (*p* < 0.05, Figure 2A,B), type III collagen (*p* < 0.05, Figure 2C,D), and fibronectin (*p* < 0.05, Figure 2E,F), compared with DMSO-treated control cells.

### 2.2. Prevention of 2-AGE-Induced Differentiation of HDFs by CB1 Antagonist

**Figure 3:**
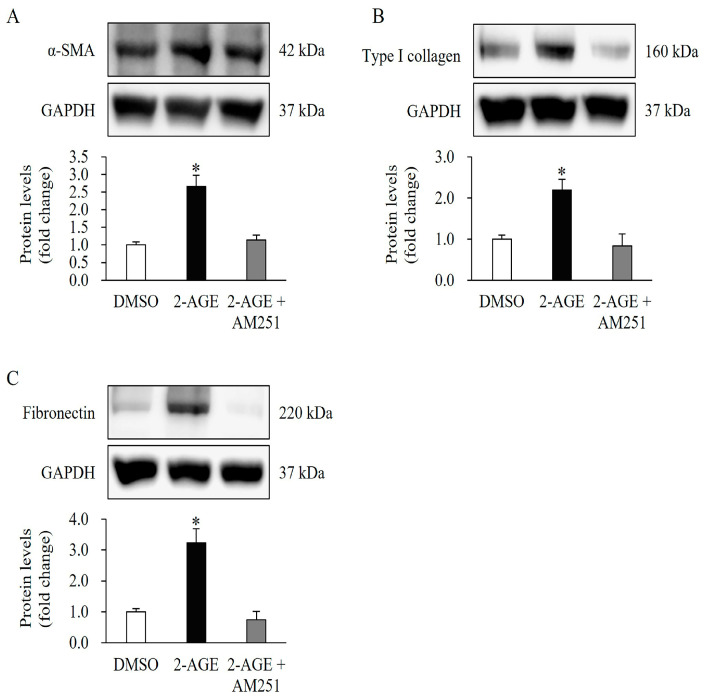
Effects of cannabinoid receptor 1 (CB1) antagonist on the expression of differentiation markers upregulated by CB1 agonists in human dermal fibroblasts (HDFs). AM251 (10 μM), a CB1-selective antagonist, was administered for 1 h prior to treatment with 2-arachidonyl glyceryl ether (2-AGE; 2.5 μM), a CB1-selective agonist, for 48 h. Western blot analysis showing that AM251 treatment abolished the upregulated expression of (**A**) alpha-smooth muscle actin, (**B**) type I collagen, and (**C**) type III collagen induced by 2-AGE treatment alone. Data are presented as mean ± SD, *n* = 3 per group. * *p* < 0.05 vs. dimethyl sulfoxide-treated control cells, which were set to 1.0.

Compared with 2-AGE (2.5 μM) treatment alone, pretreatment with AM251, a CB1-selective antagonist at 10 μM for 1 h, followed by treatment with 2-AGE at 2.5 μM, inhibited expression of α-SMA, type I collagen, and fibronectin in HDFs (*p* < 0.05, Figure 3A–C).

### 2.3. CB1 Activation Induces Epithelial-Mesenchymal Transition/Differentiation-Related Molecules and Migration in HDFs

**Figure 4:**
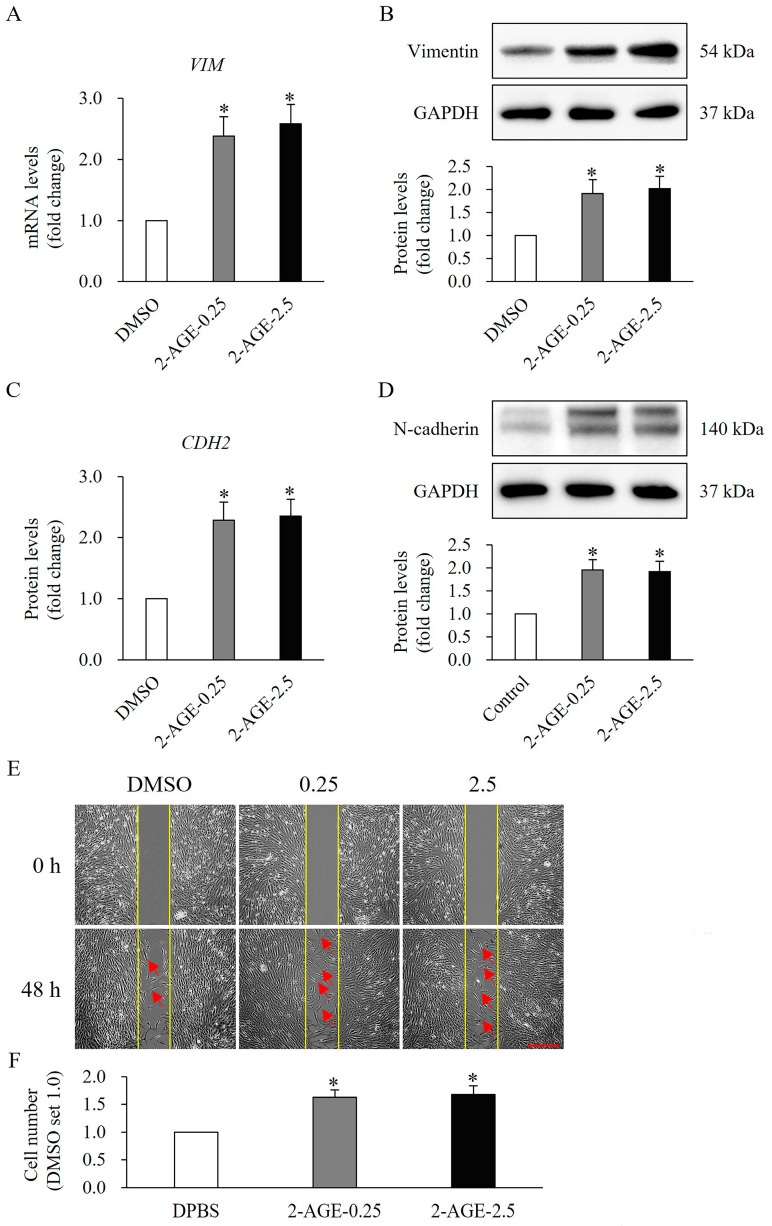
Effects of cannabinoid receptor 1 (CB1) agonist on expression of differentiation-related molecular and migration in human dermal fibroblasts (HDFs). Real-time polymerase chain reaction (PCR) analysis showing increased mRNA levels of (**A**) vimentin (*VIM*) and (**C**) N-cadherin (*CDH2*) in HDFs treated with 0.25 or 2.5 μM 2-arachidonyl glyceryl ether (2-AGE), a CB1-selective agonist, for 48 h compared with dimethyl sulfoxide (DMSO)-treated control cells. Western blot analysis showing increased protein levels of (**B**) vimentin and (**D**) N-cadherin in HDFs treated with 0.25 or 2.5 μM 2-AGE for 48 h compared with DMSO-treated cells. (**E**) Representative images of cell migration. The red arrow represents migrating cells. (**F**) Quantification of migration. Data are presented as mean ± SD, *n* = 3 per group. * *p* < 0.05 vs. DMSO-treated control cells, which were set to 1.0.

The effects of 2-AGE on HDFs differentiation were further examined by analyzing differentiation-related molecular expression and cell migration. The mRNA and protein levels of vimentin were significantly elevated following treatment with 2-AGE at 0.25 or 2.5 μM for 48 h, compared with DMSO-treated control cells (*p* < 0.05, Figure 4A,B). Similarly, both the mRNA and protein levels of N-cadherin were significantly increased after treatment with 2-AGE for 48 h, relative to DMSO-treated cells (*p* < 0.05, Figure 4C,D). Furthermore, cell migration was notably enhanced upon treatment with 2-AGE 0.25 or 2.5 μM for 48 h, compared with DMSO-treated cells (*p* < 0.05, Figure 4E,F).

### 2.4. CB1 Activation Induces SMAD and Non-SMAD Signaling in HDFs

**Figure 5:**
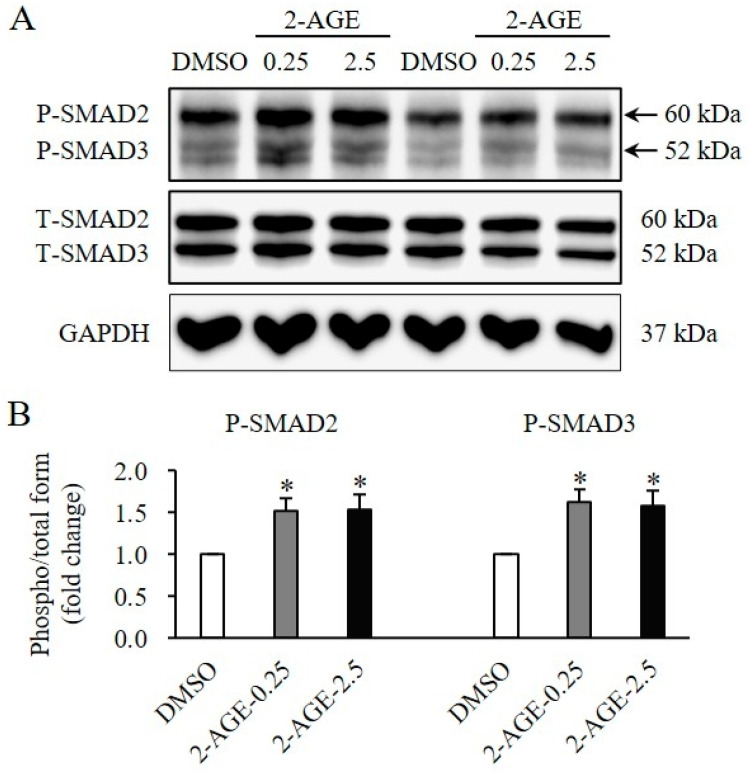
Effects of cannabinoid receptor 1 (CB1) agonist on changes of Small Mothers Against Decapentaplegic (SMAD) signaling in human dermal fibroblasts (HDFs). (**A**) Western blot analysis showing a significant increase in the phosphorylation levels of SMAD2 and SMAD3 in HDFs treated with 0.25 or 2.5 μM 2-arachidonyl glyceryl ether (2-AGE), a CB1-selective agonist, for 1 h compared to dimethyl sulfoxide (DMSO)-treated control cells. (**B**) Quantification of phosphorylated SMAD2 and SMAD3 levels. Data are presented as mean ± SD, *n* = 3 per group. * *p* < 0.05 vs. DMSO-treated control cells, which were set to 1.0.

**Figure 6:**
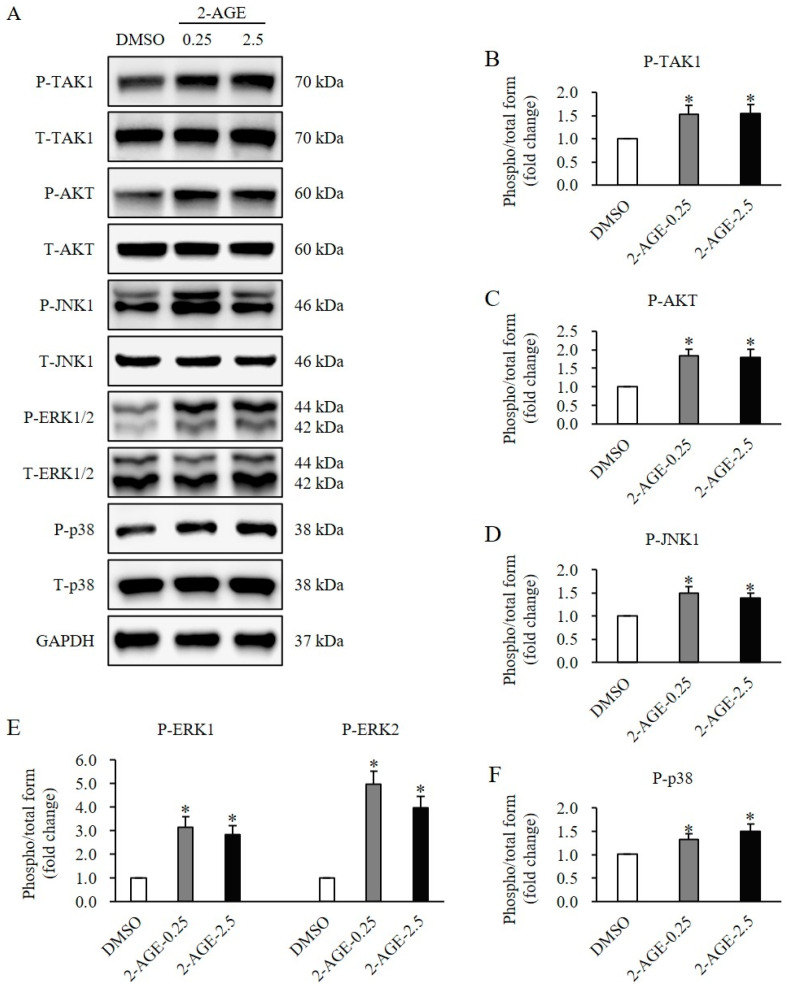
Effects of cannabinoid receptor 1 (CB1) agonist on non-Small Mothers Against Decapentaplegic (SMAD) signaling in human dermal fibroblasts (HDFs). (**A**) Western blot analysis showing a significant increase in the phosphorylation levels of TAK1, AKT, JNK1, ERK1/2 and p38 in HDFs treated with 2.5 μM 2-arachidonyl glyceryl ether (2-AGE), a CB1-selective agonist, for 1 h compared to dimethyl sulfoxide (DMSO)-treated cells. (**B**) Quantification of protein phosphorylation levels of TAK1. (**C**) Quantification of protein phosphorylation levels of AKT. (**D**) Quantification of protein phosphorylation levels of JNK1. (**E**) Quantification of protein phosphorylation levels of ERK1/2. (**F**) Quantification of protein phosphorylation levels of p38. Data are presented as mean ± SD, *n* = 3 (each group), * *p* < 0.05 vs. DMSO-treated control cells, which were set as 1.0.

To elucidate the signaling mechanisms of CB1 activation on the proliferation and differentiation of HDFs, SMAD and non-SMAD signaling pathways were assessed. The phosphorylation levels of SMAD2 and SMAD3 were significantly elevated following treatment with 2-AGE at 0.25 or 2.5 μM for 1 h, compared with DMSO-treated control cells (*p* < 0.05, Figure 5A,B). Similarly, the phosphorylation of molecules involved in non-SMAD signaling, including TAK1 (*p* < 0.05, Figure 6A,B), AKT (*p* < 0.05, Figure 6A,C), JNK1 (*p* < 0.05, Figure 6A,D), ERK1/2 (*p* < 0.05, Figure 6A,E), and p38 (*p* < 0.05, Figure 6A,F) was significantly increased following treatment with 2-AGE for 1 h, compared with DMSO-treated control cells. No significant differences were detected between 0.25 μM and 2.5 μM.

### 2.5. CB1 Activation Accelerates Wound Contraction in a Full-Thickness Mouse Wound Model

**Figure 7:**
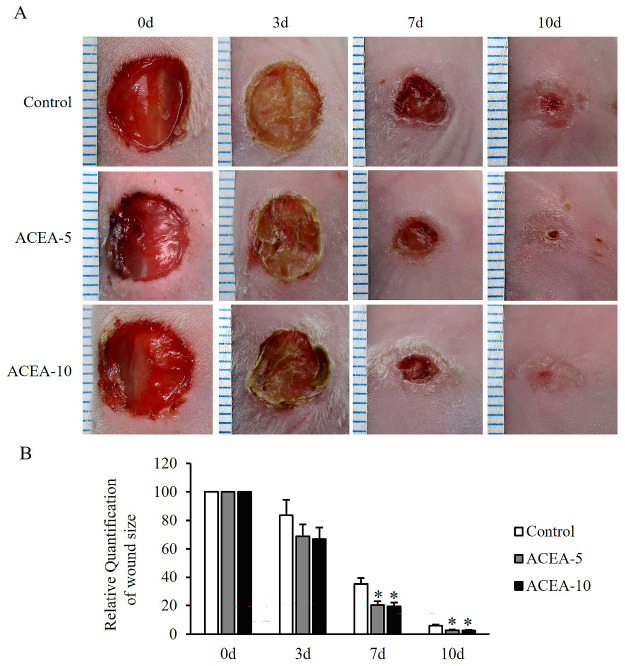
Effects of cannabinoid receptor 1 (CB1) agonist on wound contraction in a mouse full-thickness skin wound model. (**A**) Representative wound images were obtained on days 0, 3, 7, and 10 from mice treated daily with the CB1 agonist arachidonyl-2′-chloroethylamide at doses of 5 mg/kg or 10 mg/kg following injury. (**B**) Quantitative analysis of wound contraction, expressed as a percentage of the initial wound area (baseline set at 100%). Data are presented as mean ± SD, with *n* = 10 mice per group. * *p* < 0.05 vs. vehicle-treated controls, which were set at 100%.

Given the role of CB activation in HDFs, we investigated their therapeutic effects in an in vivo study. A full-thickness wound healing model was established in mice and treated with the highly selective CB1 agonist ACEA, as detailed in the Materials and Methods section. Although no statistically significant differences in wound closure were observed at day 3 after wounding, a decreasing trend was noted following treatment with ACEA at 5 or 10 mg/kg, compared to the vehicle-treated control group (Figure 7A,B). Notably, on days 7 and 10, wound closure was significantly increased, respectively, in treated groups compared with controls (*p* < 0.05, Figure 7A,B). These findings suggest that CB1 activation holds therapeutic potential for enhancing wound healing in mice.

### 2.6. CB1 Activation Enhances αSMA and ECM Expressions in Wound Tissues of a Full-Thickness Mouse Wound Model

**Figure 8:**
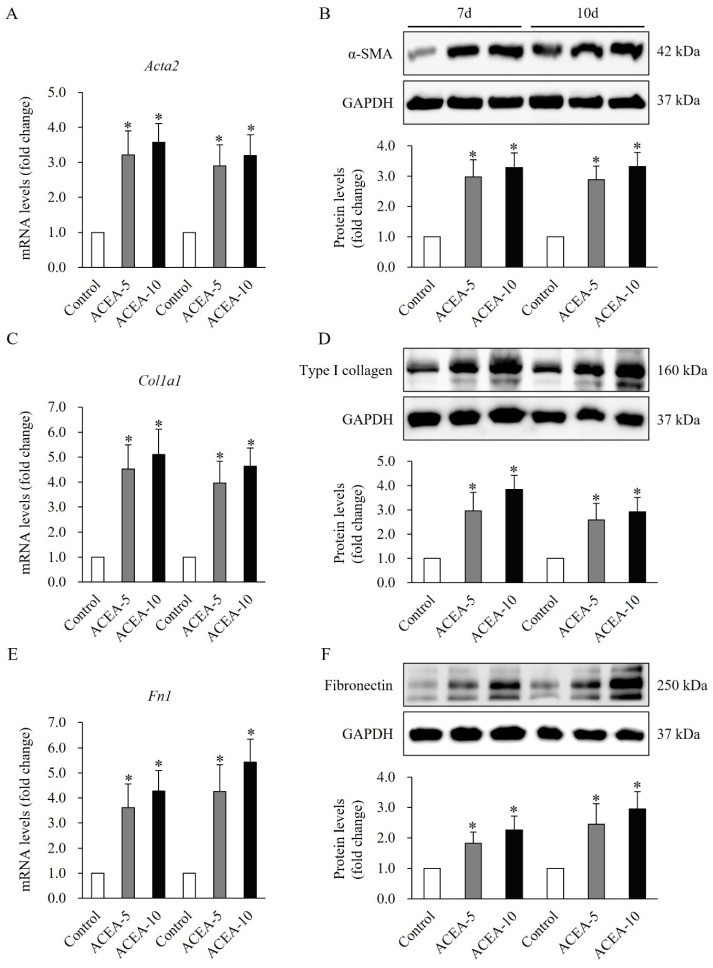
Effects of cannabinoid receptor 1 (CB1) agonist on α-smooth muscle actin (α-SMA) and extracellular matrix (ECM) expression in wound tissues of a mouse full-thickness skin wound model. Significant increase in mRNA and protein expression of α-SMA (**A**,**B**), type I collagen (**C**,**D**), type III collagen (**E**,**F**) on days 7 and 10 in wound tissues of mice administered daily with the CB1 agonist arachidonyl-2′-chloroethylamide at doses of 5 mg/kg or 10 mg/kg following injury. Data are presented as mean ± SD, with *n* = 10 mice per group. * *p* < 0.05 vs. vehicle-treated controls, which were set at 100%.

The mRNA and protein levels of αSMA were significantly elevated in wound tissues at days 7 and 10, following treatment with ACEA at 5 or 10 mg/kg, compared to the vehicle-treated control group (*p* < 0.05, Figure 8A,B). Furthermore, the mRNA and protein levels of type I collagen, a key ECM component, were significantly increased in wound tissues at days 7 and 10 following treatment with ACEA compared to the vehicle-treated control group (*p* < 0.05, Figure 8C,D). Similarly, the expression of fibronectin, another ECM component, was significantly elevated at both mRNA and protein levels in wound tissues at days 7 and 10 after treatment with ACEA, compared with the vehicle-treated control group (*p* < 0.05, Figure 8E,F). These findings suggest that CB1 activation enhances α-SMA and ECM expression, thereby promoting wound healing.

### 2.7. CB1 Activation Enhances Epidermal and Dermal Thickness as Well as Collagen Content in Wound Tissues of a Full-Thickness Mouse Wound Model

**Figure 9:**
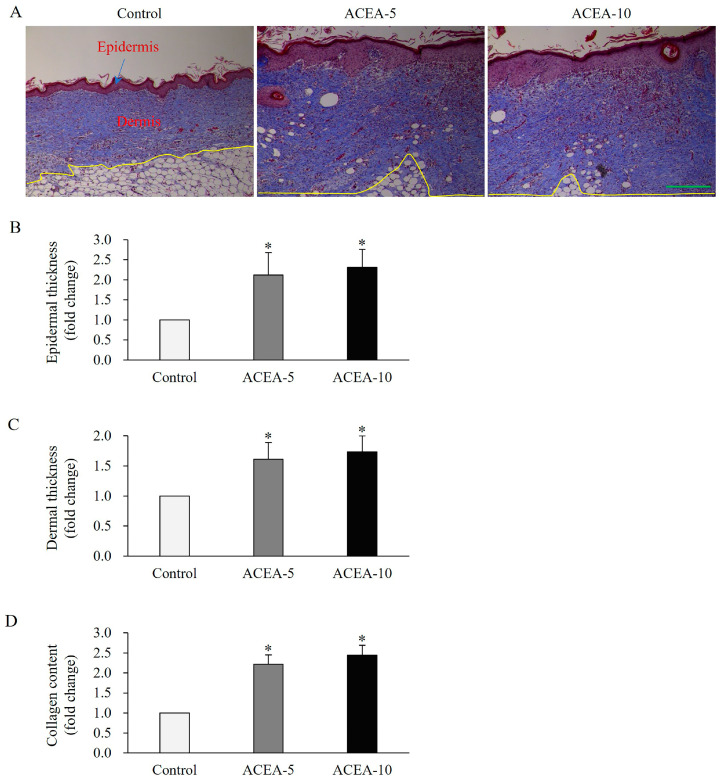
Effects of cannabinoid receptor 1 (CB1) agonist on epithelial and dermal thickness, as well as collagen deposition in wound tissues of a mouse skin full-thickness wound model. (**A**) Representative histological images by Masson’s trichrome staining on day 15 in wound tissues of mice administered daily with the CB1 agonist arachidonyl-2′-chloroethylamide at doses of 5 mg/kg or 10 mg/kg following injury. Quantitative analysis of epidermal (**B**), derm thickness (**C**), and collagen deposition (**D**). Yellow line is the boundary between dermis and subcutaneous tissues. Scale bar, 50 μm. Data are presented as mean ± SD, with *n* = 10 mice per group. * *p* < 0.05 vs. vehicle-treated controls, which were set at 1.0.

Masson’s trichrome staining was performed to evaluate epidermal and dermal thickness, as well as the collagen content in wound tissues collected on day 15 post-wounding, when the wounds were completely closed. Epidermal thickness was significantly increased following a 15-day treatment with ACEA at doses of 5 or 10 mg/kg, compared with the vehicle-treated control group (*p* < 0.05, Figure 9A,B). Similarly, dermal thickness was markedly enhanced in ACEA-treated groups compared with the controls (*p* < 0.05, Figure 9A,C). Additionally, total collagen content was significantly elevated in wound tissues after ACEA treatment compared with the vehicle-treated control group (*p* < 0.05, Figure 9A,D). These findings suggest that CB1 activation effectively promotes wound healing in a full-thickness mouse wound model.

### 2.8. CB1 Inactivation Impairs Wound Healing by Suppressing the Proliferative Phase in a Mouse Pressure Ulcer Model

CB1 activation accelerated the proliferative phase of wound healing. Therefore, we investigated whether inhibition of CB1 affects the rate of wound healing in a mouse pressure ulcer model. At day 3 post-wounding, no significant differences in wound closure were observed following treatment with the CB1-selective antagonist AM-251 or the CB2-selective antagonist AM-630 at a dose of 10 mg/kg, compared with the vehicle-treated control group (Supplementary Figure S1A,B). However, by days 7 and 10, wound closure was significantly reduced in the AM-251-treated group compared with the controls (*p* < 0.05, Supplementary Figure S1A,B), whereas no differences were observed in the AM-630-treated group. These findings suggest that CB1 inactivation decreases the rate of wound healing in mice.

α-SMA protein levels were significantly decreased in wound tissues at day 7 following treatment with AM-251 at 10 mg/kg, compared with the vehicle-treated control group (*p* < 0.05, Supplementary Figure S2A,B). Similarly, the protein levels of type I collagen and fibronectin were also significantly reduced at day 7 in the AM-251-treated group relative to controls (*p* < 0.05; Supplementary Figure S2A,B). In contrast, the expression levels of α-SMA, type I collagen, and fibronectin remained unchanged in the AM-630-treated group compared with the controls. These findings indicate that CB1 inactivation reduces α-SMA and ECM protein expression, thereby impairing wound healing.

Masson’s trichrome staining was performed to evaluate epidermal and dermal thickness, as well as the collagen content in wound tissues collected on day 15 post-wounding, when the wounds were completely closed. Epidermal thickness was significantly decreased following a 15-day treatment with CB1-selective antagonist AM-251 under 10 mg/kg, compared with the control (*p* < 0.05, Supplementary Figure S3A,B). Similarly, dermal thickness was markedly reduced in the AM-251-treated group relative to the control group (*p* < 0.05, Supplementary Figure S3A,C). Additionally, total collagen content was significantly decreased in wound tissues from AM-251-treated mice compared with the control group (*p* < 0.05, Supplementary Figure S3A,D). In contrast, epidermal and dermal thicknesses were not affected in the AM-630-treated group at a dose of 10 mg/kg, compared with the controls. These findings suggest that CB1 inactivation effectively impaired wound healing.

## 3. Discussion

The current investigation provides notable evidence, derived from both in vitro HDFs studies and an in vivo mouse model, that activation of CB1 acts as a potent molecular mechanism for promoting cutaneous wound healing. The observed acceleration of wound closure and enhanced tissue regeneration, coupled with detailed in vitro mechanistic insights, establishes CB1 activation as a promising therapeutic target for wound therapeutics.

In the present in vitro study, HDFs treatment with 2-AGE resulted in increased HDF proliferation, migration, and differentiation, which are hallmarks of activated fibroblasts during wound healing [11]. The upregulation of mesenchymal markers such as α-SMA, N-cadherin, and vimentin [12] suggests a transition toward a myofibroblast phenotype (FMT), which is critical for wound contraction and ECM remodeling [13]. Among these, as an intermediate filament protein, vimentin collaborates with the cell adhesion molecule N-cadherin to support the migratory activity of dermal fibroblasts [14,15]. Additionally, the enhanced synthesis of type I and III collagen and fibronectin indicates that CB1 activation supports ECM deposition, a key component of the proliferative and remodeling phases of wound healing [16].

Previous studies have demonstrated that activation of CB1 modulates cell proliferation across a variety of cell types. Specifically, stimulation of CB1 receptors by anandamide, an endogenous cannabinoid, has been shown to drive hepatocytes into the cell cycle [17]. In human oligodendrocytic cells, CB1 activation by ACEA and 2-AG activates signaling pathways associated with cellular proliferation, migration, and differentiation [18]. Furthermore, in vivo studies have revealed that CB1 activation enhances vascular smooth muscle cell (VSMC) proliferation [19]. Emerging evidence also suggests that CB1 activation induces fibroblast activation and contributes to fibrotic processes in various tissues, including the liver and skin [20]. For instance, CB1 stimulation has been shown to promote skin fibrosis in a CB1-dependent manner [21]. This study demonstrated that elevated CB1 activity in fibroblasts facilitates fibrosis by enhancing cell proliferation and ECM deposition. Moreover, pharmacological inhibition of CB1 attenuated fibrotic progression, further supporting its role in fibroblast activation. Consistent with these findings, our data show that the CB1 antagonist AM251 effectively suppressed 2-AGE-induced expression of α-SMA and ECM components in HDFs.

Previous studies have demonstrated that inactivation of CB1 attenuates TGF-β1-induced FMT, as evidenced by reduced α-SMA expression and collagen deposition. Notably, CB1 expression was upregulated upon TGF-β1 stimulation, suggesting its involvement in both SMAD-dependent and non-SMAD signaling pathways of the TGF-β1 axis [22]. In the present study, we mechanistically show that CB1 activation induces phosphorylation of SMAD2 and SMAD3, indicating a potential interaction with canonical TGF-β signaling, which is known to regulate fibroblast activation and fibrogenesis [23,24]. In addition to SMAD signaling, CB1 activation also engages non-SMAD pathways critically involved in cellular processes associated with wound healing. These include ERK1/2 [25,26,27], JNK [25,28,29], p38 MAPK [26,30], and AKT [28,31], all of which contribute to enhanced cell proliferation, migration, and differentiation of fibroblasts into myofibroblasts. Importantly, inhibition of TAK1, an upstream regulator of MAPK pathways, significantly suppressed fibroblast proliferation, migration, and differentiation [32,33]. Collectively, these findings suggest that CB1 signaling orchestrates a complex network of intracellular cascades, integrating both SMAD and non-SMAD pathways to regulate fibroblast activation, differentiation, and ECM synthesis during fibrotic progression.

A previous study on skin wound healing in mice revealed that CB1 expression gradually increased after injury, primarily in monocytes (MNCs) and fibroblasts (FBCs), peaking on day 5. From days 7 to 14, its expression gradually declined and was predominantly observed in FBCs [8]. These findings suggest that elevated CB1 expressions are associated with the inflammatory and proliferative phases of wound healing. Currently, the role of CB1 in inflammation is well understood in vitro and vivo. CB1 expression was increased during MNC polarization into macrophages, and in lipopolysaccharide (LPS)-treated macrophages, the CB1 blockade decreased the levels of proinflammatory mediators, including interleukin-1 beta (IL-1β), IL-6, and tumor necrosis factor-α (TNF-α) [34]. Notably, WIN55,212-2—a non-selective cannabinoid agonist—on the contrary, inhibited the production of proinflammatory cytokines in interferon gamma (IFN-γ)/LPS-treated macrophage-like THP-1 cells, but there were no different expressions of CB1 and CB2 [35]. An animal study revealed that mice with genetically deleted CB1 experienced a delay in the early stages of skin wound closure. This delayed healing is attributed to an increase in inflammatory cytokines, monocyte chemoattractant protein-1 (MCP-1), and TNF-α, secreted by CB1-deficient mesenchymal stem cells [36].

To date, evidence supporting the role of CB1 activation in animal models of skin wound healing remains limited. In the present study, we provide direct evidence of CB1-mediated wound repair, as demonstrated by both in vitro findings and an acute full-thickness wound model in mice. In vivo, treatment with the CB1 agonist ACEA significantly accelerated wound closure and upregulated the expression of αSMA, type I collagen, and fibronectin. Histological analysis revealed marked increases in both epidermal and dermal thickness, indicative of enhanced tissue regeneration. However, previous studies have shown that CB1 deficiency impairs wound healing, suggesting its functional relevance in tissue repair [36]. αSMA, a hallmark of myofibroblasts, plays a critical role in wound contraction through its ability to generate mechanical tension and draw wound margins inward. These contractile stress fibers exert force on the surrounding ECM, facilitating edge approximation and promoting tissue continuity [37]. Experimental ablation of α-SMA–positive myofibroblasts has been shown to disrupt wound healing, resulting in impaired re-epithelialization and granulation tissue formation, features commonly associated with chronic, non-healing wounds [38].

In our study, α-SMA expression was significantly elevated in fibroblasts treated with the CB1 agonist 2-AGE, as well as in wound tissues harvested on day 7 post-injury from ACEA-treated mice. This increase in α-SMA likely contributed to enhanced wound contraction observed at this time point. Furthermore, immunohistochemical analysis on day 10 revealed increased dermal thickness and collagen deposition in ACEA-treated wounds, supporting the notion that CB1 activation promotes ECM synthesis and structural remodeling.

Additional evidence from fibrotic disease models reinforces the role of CB1 in fibroblast activation. Elevated CB1 protein levels have been reported in human idiopathic pulmonary fibrosis (IPF), where CB1 overactivation contributes to disease pathogenesis. In murine models of IPF, genetic deletion or pharmacological inhibition of CB1 improved survival and reduced expression of fibrotic markers such as TGF-β1, Col1a, and Fn1 [20]. Similarly, CB1 was found to be highly expressed in kidney biopsies from patients with acute interstitial nephritis and IgA nephropathy. In a unilateral ureteral obstruction (UUO) model, blockade of CB1 significantly attenuated renal fibrosis by suppressing MCP-1 synthesis and inhibiting myofibroblast activation [39].

## 4. Materials and Methods

### 4.1. Primary Adult Normal HDFs Culture

The following experiments were conducted in strict accordance with the guidelines of the Institutional Review Board of Hallym University Hangang Sacred Heart Hospital (registration number HG2023-013) and in full compliance with the World Medical Association’s Code of Ethics (Declaration of Helsinki). A donor (male, aged 29 years) from the Burn Centre of Hallym University Hangang Sacred Heart Hospital (Seoul, Republic of Korea), who contributed to this experiment, was required to provide written informed consent prior to their participation in the study and before undergoing surgery. The primary HDFs culture was performed as previously described in our studies [40,41,42]. Tissues were obtained by 8 mm biopsy punch from donors and obligated to undergo two successive triple rinsing processes, respectively, with ethanol and cold Dulbecco’s Phosphate Buffered Saline (DPBS) (Biowest, Riverside, MO, USA). Subsequently, tissues were cut into approximately 1–2 × 1–2 mm sections, which are more conducive to digestion with dispase II solution (1 unit/mL; Gibco, Waltham, MA, USA) at 4 °C for 21 h. The dermal lay was separated from skins and further digestion was conducted in collagenase type IV solution (500 units/mL, Gibco) at 37 °C for 1 h. After inactivation with full medium, which includes Dulbecco’s modified Eagle’s medium (DMEM) enriched with 10% fetal bovine serum (FBS) (Biowest) and 1% antibiotic-antimycotic solution containing penicillin, streptomycin, and amphotericin B (Gibco), the solution was centrifuged at 1200 rpm for 5 min to acquire the cell deposit. Finally, the medium was added to T75 flasks (Eppendorf, Hamburg, Germany). The culture was sustained under controlled circumstances at 37 °C, 5% CO_2_ concentration in a cell culture incubator (Thermo Fisher Scientific, Waltham, MA, USA).

### 4.2. CB1 Agonist and Antagonist Treatment

HDFs at passage two were seeded into T75 of culture plates (Corning, Corning, NY, USA) and cultured until reaching approximately 50–60% confluency. To synchronize the cell cycle, cells were subjected to serum starvation for 24 h prior to experimental treatment. Subsequently, cells were exposed to the CB1 receptor agonist 2-AGE at concentrations of 0.25 μM or 2.5 μM. For antagonist treatment, cells were pretreated with the CB1 receptor antagonist AM251 at 10 μM (Cayman Chemical, Ann Arbor, MI, USA) for 1 h, followed by exposure to 2-AGE at 2.5 μM for 48 h. Vehicle control cells received an equivalent volume of DMSO (Sigma-Aldrich, St. Louis, MO, USA).

### 4.3. Cell Proliferation Assay

HDFs were initially seeded into 96-well plates (Corning, Corning, NY, USA) at 1 × 10^4^ cells per well. After a 2-day cell culture and a 1-day serum starvation, 2-AGE were treated for 48 h and analyzed using CellTiter-Glo Luminescent Cell Viability Assay Kit (Promega, Madison, WI, USA). Luminescence detection was conducted with a detection wavelength of 490 nm using a DTX 880 multimode detector (Beckman Coulter, Fullerton, CA, USA). The percentage calculation of cell viability is carried out by dividing (sample luminescence-background luminescence) by (control sample luminescence-background luminescence).

### 4.4. Cell Migration Assay

The cell migration assay was performed as previously described [41,42]. HDFs were initially cultured in a 2-well culture-insert system in μ-dishes (Ibidi, GmbH, Planegg, Germany) at 1 × 10^4^ cells per well and maintained for 48 h. Mitomycin C (5 μg/mL; Sigma, St. Louis, MO, USA) being added 6 h to eliminate the influence of cell proliferation prior starting cell migration. Next, inserts between the 2 wells were removed (indicating starting migration) and 0.25 or 2.5 μM of 2-AGE was added. Cell images were separately captured at time points of 0- and 48-h treatment using light microscopy (IX 70, Olympus, Tokyo, Japan). The supporting quantitative analysis of the experimental through ImageJ software (1.54p, National Institutes of Health, Bethesda, MD, USA).

### 4.5. Animal Wound Models and Administration

The animal study was approved by the Animal Research Ethics Board of Hallym University (registration number HMC 2022-2-0525-27, 2022-7-1) and conducted in full compliance with the National Institutes of Health’s Guide for the Care and Use of Laboratory Animals. Nude male mice were obtained from Koatech Laboratory Animal Center (Pyeongtaek, Gyeonggi-do, Republic of Korea). These mice were specifically prepared to meet the criteria of 6 weeks of age and weighing approximately 18–20 g. They were housed in the Specific Pathogen-Free (SPF) room of the animal center at the Ilsong Institute of Life Science, Hallym University. Mice were randomly assigned to 3 groups with 10 mice per group, kept in polycarbonate cages under controlled conditions: room temperature of 25 ± 2 °C, relative humidity of 55 ± 5%, and 12/12-h light/dark cycle. They were provided with standard laboratory chows and filtered water. After 2 weeks, the full-thickness wound model was prepared as described previously [43]. Mice were anesthetized and maintained with 2.5% isoflurane (Hana Pharm, Seoul, Republic of Korea) in 100% oxygen. A full-thickness dorsal wound measuring 10 mm in diameter was created on each mouse using a 10-mm dermal biopsy punch (Acuderm, Fort Lauderdale, FL, USA). The potent and highly selective CB1 agonist arachidonyl-2′-chloroethylamide (ACEA) [9] was dissolved in a mixture of 30% PEG400, 0.5% Tween 80, 5% propylene glycol, and 64.5% (*wt*/*wt*) distilled water [44] to prepare concentrations of 2.5 mg/mL and 5 mg/mL, respectively. A volume of 50 μL corresponding to 5 or 10 mg/kg of ACEA [9,45] was injected subcutaneously (S.C.) around the wounds twice daily for 15 days. The sham control group received the same solvent mixture without the active drugs. After wounding, daily dressing was performed using Tegaderm™ (3M, Saint Paul, MN, USA) until the wound fully healed.

### 4.6. Measurement of Wound Closure

To analyze the wound size, images were captured using a digital camera (Nikon, Tokyo, Japan) on days 0, 3, 7, and 10 after the wound was created. The quantification of wound closure was performed using ImageJ software (National Institutes of Health, Bethesda, MD, USA), normalized to the wound size at day 0, which was set as 100% as described in our previously published study [43].

### 4.7. Reverse Transcription–Quantitative Polymerase Chain Reaction (RT-qPCR)

Harvested cells were dissolved in Trizol (Invitrogen, Carlsbad, CA, USA) and wound tissues were homogenized in Trizol using the gentleMACS Dissociator (Miltenyi Biotect, Bergisch-Gladbach, Germany). Total RNA was extracted with a ReliaPrep™ RNA Miniprep system (Promega, Madison, WI, USA), followed by the use of nanodrop spectrophotometer (BioTek Instruments Inc., Winooski, VT, USA) to determine its concentration. RNA was reverse transcribed into cDNA through PrimeScript™ RT master mix (Takara Bio Inc., Kusatsu, Japan). A 20-μL reaction system was utilized encompassing cDNA, 2xPCR premix (Takara, Shiga, Japan), and primers (Origene, Rockville, MD, USA). Primer sequences are presented in Table 1 [40]. RT-qPCR was run in a Light Cycler 96 system (Roche, Basel, Switzerland). Considering GAPDH as normalization reference, received data were quantified through the 2^−ΔΔ^Ct method to analyze the expression levels of genes.

### 4.8. Western Blot

Harvested cells were lysed and added to Radio-Immunoprecipitation Assay (RIPA) buffer supplemented with protease and phosphatase inhibitors (Sigma, St. Louis, MO, USA). After centrifugation for 30 min at 15,000 rpm, supernatants were collected and the protein concentration was ascertained using the BCA kit (Thermo Fisher Scientific) in accordance with the manufacturer’s instructions; further, 5× SDS-PAGE protein loading buffer (Cell Signaling Technology, Danvers, MA, USA) was added and boiled. To conduct electrophoresis, 8% of SurePAGE™ Bis-Tris gel (GenScript, Piscataway, NJ, USA) was judiciously selected in accordance with the size of the protein. The gels electro-transfer onto polyvinylidene difluoride membrane (PVDF, 0.45 μm pore size) (Millipore, Billerica, MA, USA), and blocked with 5% skim milk (BD DIFCO, Franklin Lakes, NJ, USA) in 1× Tris-buffered saline with Tween-20 (TBST) buffer. Upon incubation with the primary antibodies overnight, in accordance with Table 2 at 4 °C, the samples were washed thrice using TBST. Upon incubation with the secondary antibodies (horseradish peroxidase-conjugated goat anti-rabbit or anti-mouse IgG; Millipore) diluted at a concentration of 1:2500 for 1 h, they were washed another three times using TBST. The freshly prepared ECL luminescence solution (ATTO, Tokyo, Japan) was used to capture the images of Western blot bands through the chemiluminescence imaging system (WSE-6100; ATTO) for visualization collection. With GAPDH as a reference for normalization, the grayscale was analyzed by ImageJ software (National Institutes of Health).

### 4.9. Histology and Measurement of Epithelial and Dermal Thickness

Wound tissues were harvested on day 15 after wounding. Tissues were fixed in 4% formaldehyde, preserved at 25 °C overnight, and consecutively immersed in ethanol of gradient concentrations ranging from 70% to 100% for dehydration. Subsequently, alcohol was removed by xylene, and tissue was embedded in paraffin. Paraffin blocks were sliced into 5-μm sections, flattened and adhered onto microscope slide (Muto Pure Chemicals, Tokyo, Japan). Sections were dewaxed, hydrated, and respectively stained with Masson’s trichrome (Cancer Diagnostics, Inc., Durham, NC, USA) according to the manufacturer’s instructions, followed by dehydration and clarification. The mounting solution (Cancer Diagnostics, Inc., Durham, NC, USA) was added dropwise to seal the slides, and morphological alterations were observed and recorded at a magnification of 20× using a light microscope (Eclipse Si, Nikon, Tokyo, Japan). The following procedures are carried out as described previously [46]. The maximum and minimum values of epidermal and dermal thickness, as well as intensity, were measured using ImageJ software (National Institutes of Health, Bethesda, MD, USA; Fiji, version 2.16.0). Normalization was performed by setting the solvent mixture-treated sham group as the control (value of 1.0).

### 4.10. Statistical Analysis

Data are expressed as means ± standard deviation (SD). Statistical analysis was performed using SPSS Statistics (version 24.0; SPSS, Inc., Chicago, IL, USA). Group distributions were assessed using the Kruskal–Wallis test, a nonparametric alternative to one-way analysis of variance (ANOVA). When significant differences were detected, post-hoc comparisons between two groups were conducted using the Mann–Whitney U test with Bonferroni correction. Statistical significance was set at *p* < 0.05 or *p* < 0.01.

## 5. Conclusions

In conclusion, our findings support the hypothesis that CB1 receptor activation facilitates wound healing through both cellular and molecular mechanisms. The dual engagement of SMAD and non-SMAD pathways underscores the complexity of CB1-mediated signaling and its potential to modulate multiple aspects of tissue repair. Thus, these findings strongly support the therapeutic potential of targeting specific agonists as a viable strategy to accelerate the proliferative or contractile phases and thereby enhance the rate of wound healing. Future research should focus on optimizing agonist delivery (e.g., topical formulations), exploring its efficacy in models of chronic, non-healing wounds (e.g., diabetic ulcers), and investigating the impact of activation on mitigating adverse scarring outcomes.

## Figures and Tables

**Table 1:** Real-time PCR primer sequences.

Gene	Forward (5′ → 3′)	Reverse (5′ → 3′)
*GAPDH (h)*	CATGAGAAGTATGACAACAGCCT	AGTCCTTCCACGATACCAAAGT
*ACTA2 (h)*	CCGACCGAATGCAGAAGGA	ACAGAGTATTTGCGCTCCGAA
*FN1 (h)*	CCAGTCCACAGCTATTCCTG	ACAACCACGGATGAGCTG
*COL1A1 (h)*	ATGTTCAGCTTTGTGGACCTC	CTGTACGCAGGTGATTGGTG
*COL3A1 (h)*	CACTGGGGAATGGAGCAAAAC	ATCAGGACCACCAATGTCATAGG
*CDH2 (h)*	ACCGACACTCCTACAAGATTT	GCAGAAACAAGTTGGTTGGATA
*VIM (h)*	GTCAGAACTAAAGGAGCTGC	TGTTGCTGTCCAAGTTGCTC
*Acta2 (m)*	CAGATGTGGATCAGCAAACAGGA	GACTTAGAAGCATTTGCGGTGGA
*Col1a1 (m)*	GACATGTTCAGCTTTGTGGACCTC	GGGACCCTTAGGCCATTGTGTA
*Fn1 (m)*	ATCATAGTGGAGGCACTGCAGAA	GGTCAAAGCATGAGTCATCTGTAGG

**Table 2:** Primary antibodies used in Western blot analysis.

Target	Host	Dilution	Company (Cat. No.)
GAPDH	Rabbit	1:1000	Cell Signaling Technology (2118S)
GAPDH	Mouse	1:1000	Santa Cruz Technology, Dallas, TX, USA (sc-47724)
α-SMA	Mouse	1:500	Abcam, Cambridge, MA, USA (ab7817)
Fibronectin	Rabbit	1:2000	Abcam (ab6328)
Collagen I	Rabbit	1:1000	Abcam (ab34710)
Collagen III	Rabbit	1:1000	Abcam (ab7778)
Vimentin	Mouse	1:3000	Abcam (ab92547)
N-Cadherin	Mouse	1:1000	Invitrogen (333900)
Phospho-SMAD2/3	Rabbit	1:1000	Cell Signaling Technology (8828S)
SMAD2/3	Rabbit	1:1000	Cell Signaling Technology (3102S)
Phospho-TAK1	Rabbit	1:1000	Cell Signaling Technology (9339S)
TAK1	Rabbit	1:1000	Cell Signaling Technology (5206S)
Phospho-p38	Mouse	1:1000	Cell Signaling Technology (9216S)
p38	Rabbit	1:1000	Cell Signaling Technology (8690S)
Phospho-JNK	Rabbit	1:1000	Cell Signaling Technology (9251S)
JNK	Rabbit	1:1000	Cell Signaling Technology (9252S)
Phospho-ERK	Rabbit	1:1000	Cell Signaling Technology (4370S)
ERK	Mouse	1:1000	Cell Signaling Technology (4696S)
Phospho-AKT	Rabbit	1:1000	Cell Signaling Technology (4060S)
AKT	Rabbit	1:1000	Cell Signaling Technology (4671S)

## Data Availability

The original contributions presented in this study are included in the article/Supplementary Materials. Further inquiries can be directed to the corresponding author.

## Supplementary Materials

The following supporting information can be downloaded at https://www.mdpi.com/article/10.3390/ijms27031171/s1. References [47,48,49,50] are cited in the Supplementary Materials.

## Abbreviations

The following abbreviations are used in this manuscript:

HTS	hypertrophic scar
ECM	extracellular matrix
FMT	fibroblast-to-myofibroblast transition
IPF	idiopathic pulmonary fibrosis
UUO	unilateral ureteral obstruction
CB1	cannabinoid receptor 1
THC	tetrahydrocannabinol
2-AGE	2-arachidonyl glyceryl ether
ACEA	arachidonoyl 2′-chloroethylamide
ECS	endocannabinoid system
TCBM	topical application of Cannabis-Based Medicine
LPS	lipopolysaccharide
HDF	human dermal fibroblast
VSMC	vascular smooth muscle cell
MNC	monocyte
FBC	fibroblast
TGF-β1	transforming growth factor beta 1
α-SMA	alpha-smooth muscle actin
BMP	bone morphogenetic protein
TNF-α	tumor necrosis factor-α
IL-1β	interleukin-1 beta
IFN-γ	interferon gamma
MCP-1	monocyte chemoattractant protein-1
TAK1	activated kinase 1
JNK	Jun terminal kinase
ERK1/2	extracellular signal-regulated kinase 1/2
AKT	protein kinase B
NF-κB	nuclear factor kappa-light-chain-enhancer of activated B cells
SPF	Specific Pathogen-Free
DPBS	Dulbecco’s Phosphate Buffered Saline
FBS	fetal bovine serum
