# Supplementary material for: Activation of Cannabinoid Receptor 1 Enhances Wound Healing by Promoting the Proliferative Phase
Source: Int J Mol Sci. 2026 Jan 23;27(3):1171. doi: 10.3390/ijms27031171 (PMC12897412; doi:10.3390/ijms27031171)
Supplement: Supplementary file 1 [file ijms-27-01171-s001.zip › ijms-4082296-supplementary.pdf]

Supplemental information

Activation of cannabinoid receptor 1 enhances wound healing by promoting the proliferative phase

Hui Song Cui [1 †], Ya Xin Zheng [1 †], Yoon Soo Cho [2], Yeon Gyun Jung[1], In Suk Kwak [3], Yu Mi Ro[1], So Young Joo [2], June-Bum Kim [4*], Cheong Hoon Seo [2 *]

Supplementary Materials and Methods

Hairless male mice (8 weeks old) were obtained from Koatech Laboratory Animal Center (Pyeongtaek, Gyeonggi-do, Republic of Korea). All animal experiments were conducted in the animal facility of the Ilsong Institute of Life Science, Hallym University, in accordance with the Guidelines for the Care and Use of Laboratory Animals of the National Institutes of Health. Experimental protocols were approved by the Animal Research Ethics Board of Hallym University (registration number HMC 2021-2-0614-27). Mice were randomly assigned to three groups (10 mice per group) and housed in polycarbonate cages under controlled conditions: room temperature of 25 ± 2 °C, relative humidity of 55 ± 5%, and a 12/12-h light/dark cycle. Standard laboratory chow and filtered water were provided ad libitum. After 2 weeks of acclimatization, mice were anesthetized with 2.5% isoflurane in 100% oxygen (Hana Pharm, Seoul, Korea). The dorsal skin was sterilized with 70% ethanol, gently elevated, and loaded with two circular ceramic magnetic plates (1.2 g, 1500 Gauss, 8.0 mm diameter, 2.0 mm thickness), creating a 5.0 mm-thick skin bridge between the magnets. The magnets were applied for 12 h and removed for 12 h, constituting a single ischemia–reperfusion (I/R) cycle. After three cycles, a complete pressure ulcer (PU) was established [1,2]. The selective CB1 antagonist AM-251 and CB2 antagonist AM-630 were dissolved in a vehicle consisting of 30% PEG400, 0.5% Tween 80, 5% propylene glycol, and 64.5% distilled water (w/w) to prepare 5 mg/mL solutions. A volume of 50 μL, corresponding to 10 mg/kg of AM-251 or AM-630 [3,4], was injected

subcutaneously around the wounds twice daily for 15 days. The sham control group received the same vehicle without active compounds. Following wounding, daily dressings were applied using Tegaderm™ (3M, Saint Paul, MN, USA) until complete wound closure was achieved. Measurements of wound closure, protein expression by western blot, histological analysis, and assessments of epithelial and dermal thickness were performed as described in the Materials and Methods section of the manuscript.

References

1. Lanzafame, R.J.; Stadler, I.; Cunningham, R.; Muhlbauer, A.; Griggs, J.; Soltz, R.; Soltz, B.A. Preliminary assessment of photoactivated antimicrobial collagen on bioburden in a murine pressure ulcer model. Photomed Laser Surg 2013, 31, 539-546, doi:10.1089/pho.2012.3423.

2. Cui, H.S.; Lee, Y.R.; Ro, Y.M.; Joo, S.Y.; Cho, Y.S.; Kim, J.B.; Kim, D.H.; Seo, C.H. Knockdown of CPEB1 and CPEB4 Inhibits Scar Formation via Modulation of TAK1 and SMAD Signaling. Ann Dermatol 2023, 35, 293-302, doi:10.5021/ad.22.210.

3. Parihar, V.K.; Syage, A.; Flores, L.; Lilagan, A.; Allen, B.D.; Angulo, M.C.; Song, J.; Smith, S.M.; Arechavala, R.J.; Giedzinski, E.; et al. The Cannabinoid Receptor 1 Reverse Agonist AM251 Ameliorates Radiation-Induced Cognitive Decrements. Front Cell Neurosci 2021, 15, 668286, doi:10.3389/fncel.2021.668286.

4. Onaivi, E.S.; Carpio, O.; Ishiguro, H.; Schanz, N.; Uhl, G.R.; Benno, R. Behavioral effects of CB2 cannabinoid receptor activation and its influence on food and alcohol consumption. Ann N Y Acad Sci 2008, 1139, 426-433, doi:10.1196/annals.1432.035.

Results

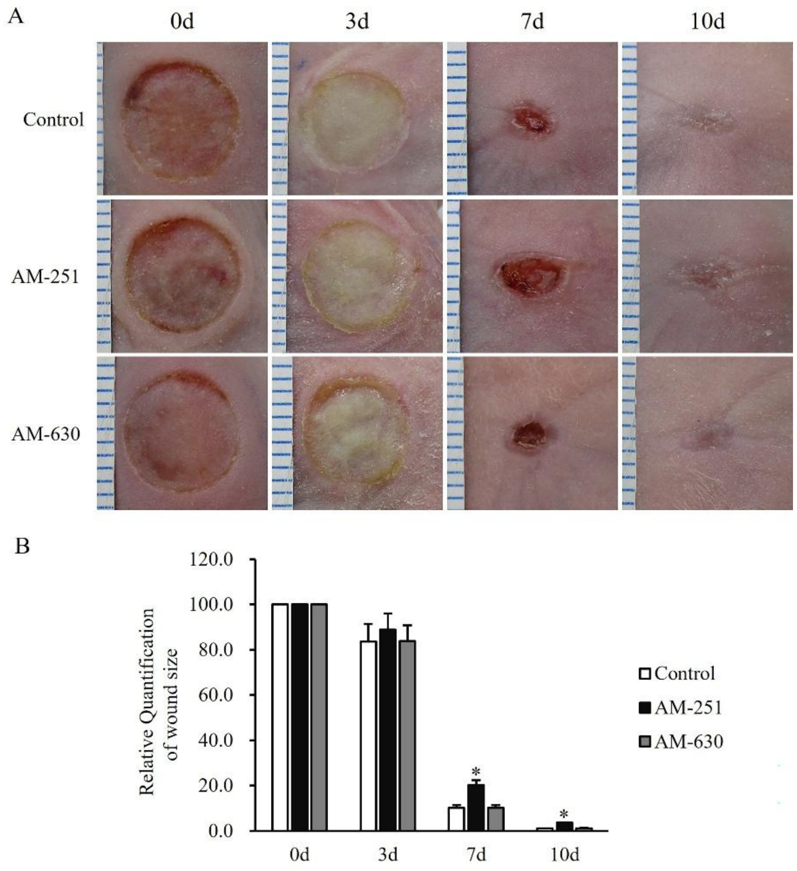

Supplementary Figure 1. Effects of CB1 and CB2 antagonists on wound contraction in a mouse pressure ulcer model. (A) Representative wound images were obtained on days 0, 3, 7, and 10 from mice treated daily with the CB1-selective antagonist AM-251 or CB2-selective antagonist AM-630 at a dose of 10 mg/kg following injury. (B) Quantitative analysis of wound contraction, expressed as a percentage of the initial wound area (baseline set at 100%). Data are presented as mean ± SD, with n=10 mice per group. *$P < 0.05$ vs. vehicle-treated controls, which were set at 100%.

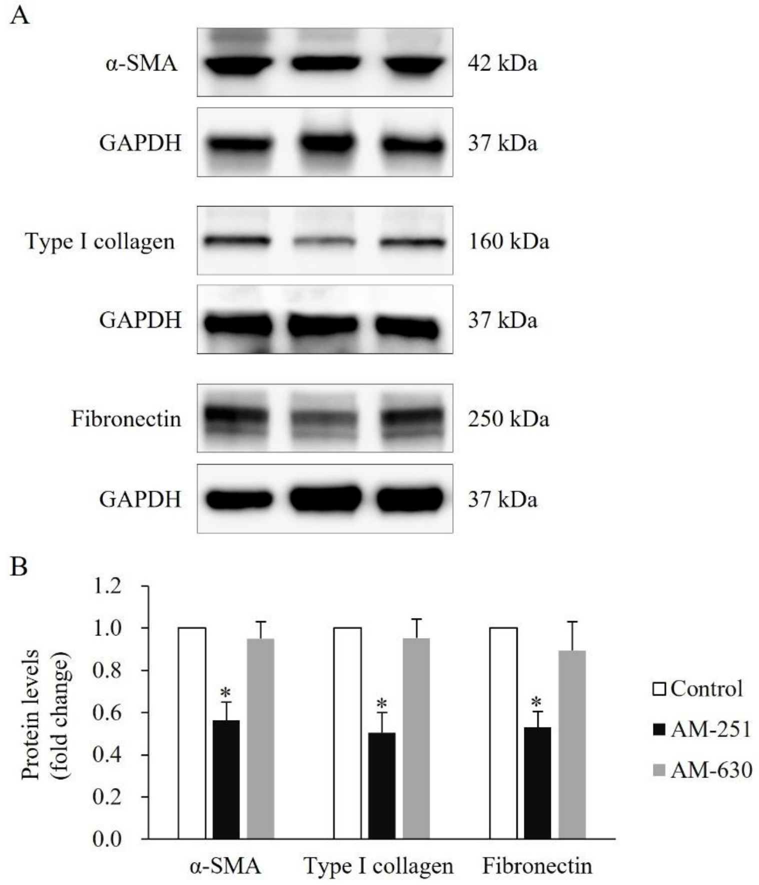

Supplementary Figure 2. Effects of CB1 and CB2 antagonists on α-SMA and ECM expression in wound tissues of a mouse pressure ulcer model. Western blot analysis showing a significant decrease in protein expression of α-SMA, type I collagen, and fibronectin on day 7 in wound tissues of mice administered daily with the CB1-selective antagonist AM-251 at a dose of 10 mg/kg following injury. Data are presented as mean ± SD, with n=10 mice per group. *P < 0.05 vs. vehicle-treated controls, which were set at 100%.

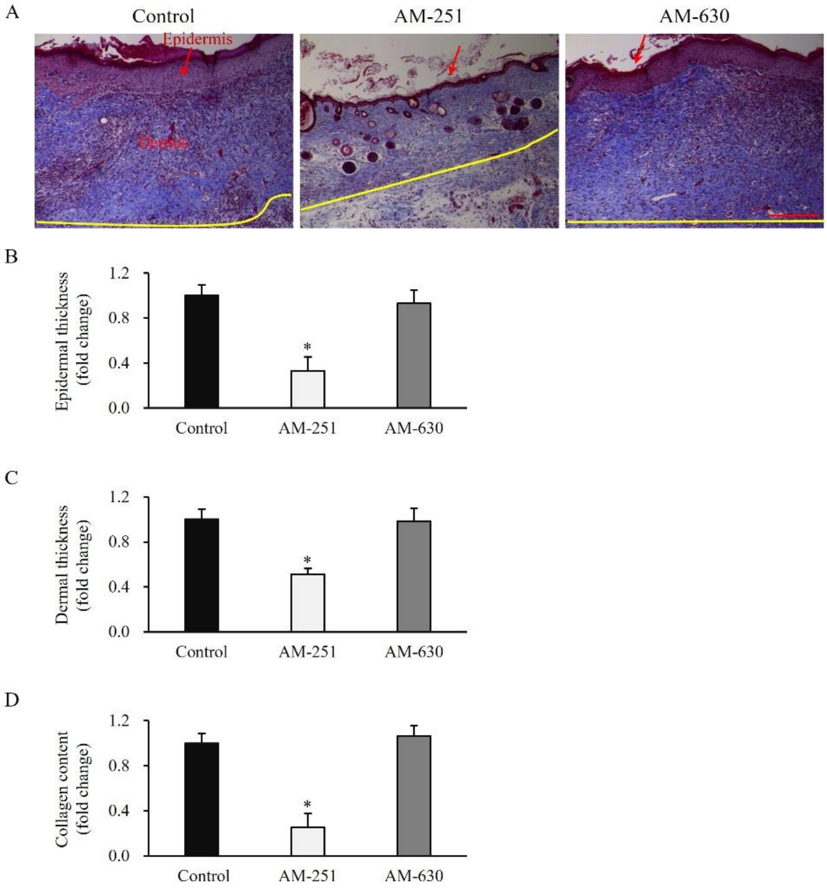

Supplementary Figure 3. Effects of CB1 and CB2 antagonists on epithelial and dermal thickness, as well as collagen deposition in wound tissues of a mouse pressure ulcer model. (A) Representative histological images by Masson's trichrome staining on day 15 in wound tissues of mice administered daily with the CB1-selective antagonist AM-251 or the CB2-selective antagonist AM-630 at a dose of 10 mg/kg following injury. Quantitative analysis of epi-dermal (B), derm thickness (C), and collagen deposition (C). Scale bar, 50 μm. Data are presented as mean ± SD, with n=10 mice per group. *$P < 0.05$ vs. vehicle-treated controls, which were set at 1.0.
